# Alcohol consumption during pregnancy differentially affects the fecal microbiota of dams and offspring

**DOI:** 10.1038/s41598-024-64313-z

**Published:** 2024-07-12

**Authors:** Tamara S. Bodnar, Garrett Ainsworth-Cruickshank, Vincent Billy, Laura Wegener Parfrey, Joanne Weinberg, Charlis Raineki

**Affiliations:** 1 Department of Cellular and Physiological Sciences, University of British Columbia, Vancouver, BC Canada; 2 Department of Biological Sciences, University of Calgary, 2500 University Dr NW, Calgary, AB T2N 1N4 Canada; 3 Department of Zoology and Biodiversity Research Centre, University of British Columbia, Vancouver, BC Canada; 4 Department of Botany, University of British Columbia, Vancouver, BC Canada; 5 Department of Psychology, Brock University, St. Catharines, ON Canada

**Keywords:** Microbiome, Disease model

## Abstract

Microbiota imbalances are linked to inflammation and disease, as well as neurodevelopmental conditions where they may contribute to behavioral, physiological, and central nervous system dysfunction. By contrast, the role of the microbiota in Fetal Alcohol Spectrum Disorder (FASD), the group of neurodevelopmental conditions that can occur following prenatal alcohol exposure (PAE), has not received similar attention. Here we utilized a rodent model of alcohol consumption during pregnancy to characterize the impact of alcohol on the microbiota of dam-offspring dyads. Overall, bacterial diversity decreased in alcohol-consuming dams and community composition differed from that of controls in alcohol-consuming dams and their offspring. Bacterial taxa and predicted biochemical pathway composition were also altered with alcohol consumption/exposure; however, there was minimal overlap between the changes in dams and offspring. These findings illuminate the potential importance of the microbiota in the pathophysiology of FASD and support investigation into novel microbiota-based interventions.

## Introduction

There is growing recognition of the wide ranging influence of the gut microbiota on physiological function, with alterations linked to a variety of disease states (reviewed in Ref.^1^). Dysbiosis, or imbalances in the gut microbial community, can lead to disruptions in intestinal barrier function, and increased bacterial components in circulation, resulting in inflammation and disease^2–4^. The impact of the microbiota also extends to the central nervous system, with microbiota alterations linked to altered neurodevelopment^5^, behavior^6^ and a wide variety of neuropsychiatric conditions^7^. This increasing appreciation for the extensive influence of the microbiota has led to investigations into the role of the microbiota in the pathophysiology of neurodevelopmental disorders^5^. Specifically, alterations in the gut microbiota have been detected in autism spectrum disorder (ASD)^8^, attention-deficit/hyperactivity disorder (ADHD)^9^, and schizophrenia^10^. By contrast, the role of microbiota disturbances in the pathophysiology of Fetal Alcohol Spectrum Disorder (FASD), the group of neurodevelopmental conditions that occur as a result of in utero alcohol exposure, has not received similar research attention. Recent work from our group using a rodent model showed, for the first time, that prenatal alcohol exposure (PAE) results in long-lasting changes in the microbiota, including increased richness of bacterial species and community structure differences in adulthood^11^; however, more work is urgently needed in this area.

It has been well-established that alcohol consumption in *adulthood* impacts the microbiota (reviewed in Refs.^12,13^). Overall, adult alcohol consumption results in a wide range of effects including: impaired intestinal barrier function^14^, increased gut permeability, resulting in bacterial translocation^15^, as well as dysbiosis^16^, and increased abundance of pro-inflammatory gut bacteria^17^. By contrast, much less is known regarding the impact of alcohol-consumption on the pregnant organism and her offspring. Pregnancy, in general, is characterized by profound shifts in the gut microbiota over time, including reduced bacterial richness, to help support fetal development^18^. And based on the limited data available, alcohol-consumption during pregnancy appears to further alter the gut microbiota. Specifically, evidence from a pre-clinical study suggests that taxa including clostridium and helicobacter are increased in alcohol-consuming pregnant rats^19^ and a small clinical study (alcohol-consuming group, n = 10) found altered taxa in alcohol-consuming mothers and their newborns^20^. Taken together, the extensive impact of alcohol on gut function and microbiota composition in the adult organism, and the suggestion that alcohol consumption during pregnancy may further alter the microbiota, lends support to the idea that PAE may also have a significant impact on gut structure and microbiota composition.

A key open question is whether PAE alters the microbiota through direct transmission of the altered maternal microbiota or indirectly via alteration of gut or neuroimmune development. As colonization of the offspring is seeded by the maternal microbiota through vertical transmission mainly at birth and during early life^21^, a key question remains, in the context of in utero alcohol exposure—is the alcohol-affected maternal microbiota signature detectable in the offspring? Or, given that developmental exposure to alcohol occurs during gut development and prior to gut closure^22^, are there other factors critically influencing and causing divergence of microbiota in the offspring? To begin to answer this question, the current study examined the fecal microbiota in dam-offspring dyads, including alcohol-consuming dams and their PAE offspring, as well as control counterparts. Building on our previous work^11^, we hypothesized that both alcohol-consumption during pregnancy and PAE would alter bacteria at the community level by impacting the overall number of unique taxa (richness), and their relative abundance (evenness). Additionally, we expected the overall bacterial community structure to shift; however, we predicted minimal overlap in alcohol-induced changes between the dam and offspring due to the developmental timing of the alcohol exposure.

## Results

### Alcohol consumption during pregnancy and PAE both affect microbial community structure

To examine the impact of alcohol consumption during pregnancy on the maternal and offspring fecal microbiota, community level analyses of bacterial richness (α diversity) and community structure (β diversity) were performed on fecal samples collected on gestational day 14 (GD14) and postnatal day 22 (P22), for the dams and offspring, respectively.

**Figure 1:**
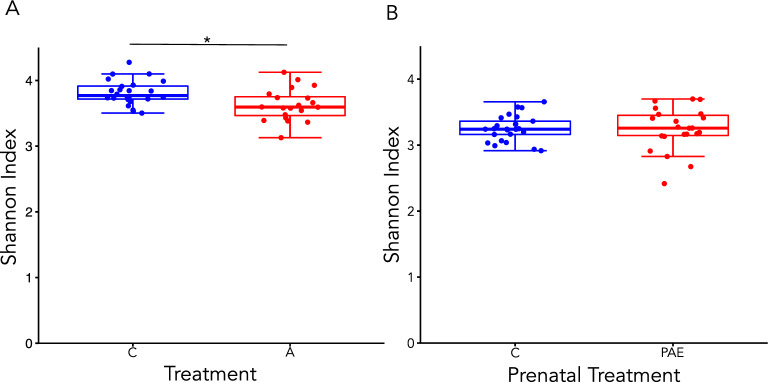
Bacterial richness and evenness (α-diversity). Boxplots displaying the Shannon index (α-diversity) for dams (**A**) and offspring (**B**). *p < 0.05; A alcohol, PAE prenatal alcohol exposure, C control. A dams: n = 20; C dams = 24; PAE offspring: n = 22; C offspring: n = 25.

α-diversity was assessed using the Shannon index, which measures bacterial richness and evenness. The Shannon index was lower in alcohol-consuming dams compared to controls (t(35.7) = 2.76, p = 0.01; Fig. 1A). By contrast, there were no differences in the Shannon index between PAE and control offspring (t(33.7) = 0.16, p = 0.88; Fig. 1B).

**Figure 2:**
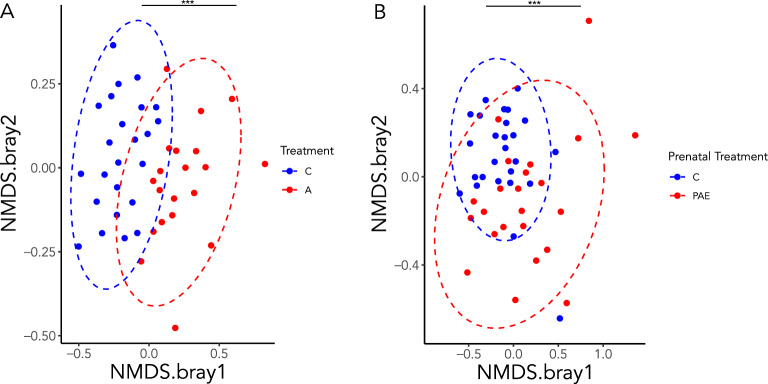
Bacterial community structure (β-diversity). Non-metric multidimensional scaling (NMDS) plots of Bray–Curtis dissimilarity with ellipsoids representing 95% confidence intervals for dams (**A**) and offspring (**B**). ***p < 0.001; A alcohol, PAE prenatal alcohol exposure, C control. A dams: n = 20; C dams = 24; PAE offspring: n = 23; C offspring: n = 26.

Community structure (β diversity) was compared across groups using the Bray–Curtis dissimilarity index (which considers presence and absence and relative abundance), and visualized using non-metric multidimensional scaling (NMDS) plots. For the dams, alcohol consumption during pregnancy resulted in distinct community composition compared to that in controls (PERMANOVA: Pseudo-F = 8.14, df = 1, R^2^ = 0.16, p = 0.001), with distinct clustering apparent in the NMDS plots (Fig. 2A). Similarly, distinct clustering was observed between PAE and control offspring, and community composition again differed significantly (PERMANOVA: Pseudo-F = 5.20, df = 1, R^2^ = 0.10, p = 0.001; Fig. 2B).

Sex differences in bacterial community richness and evenness and community composition were also explored for the offspring. However, interactions between prenatal treatment and sex were not identified (α-diversity: Shannon: F(1,43) = 2.22, p = 0.14; β-diversity: Bray–Curtis: Pseudo-F = 0.38, df = 1, R2 = 0.01, p = 0.96; Supplementary Materials) and therefore subsequent analyses were conducted on males and females combined.

**Figure 3:**
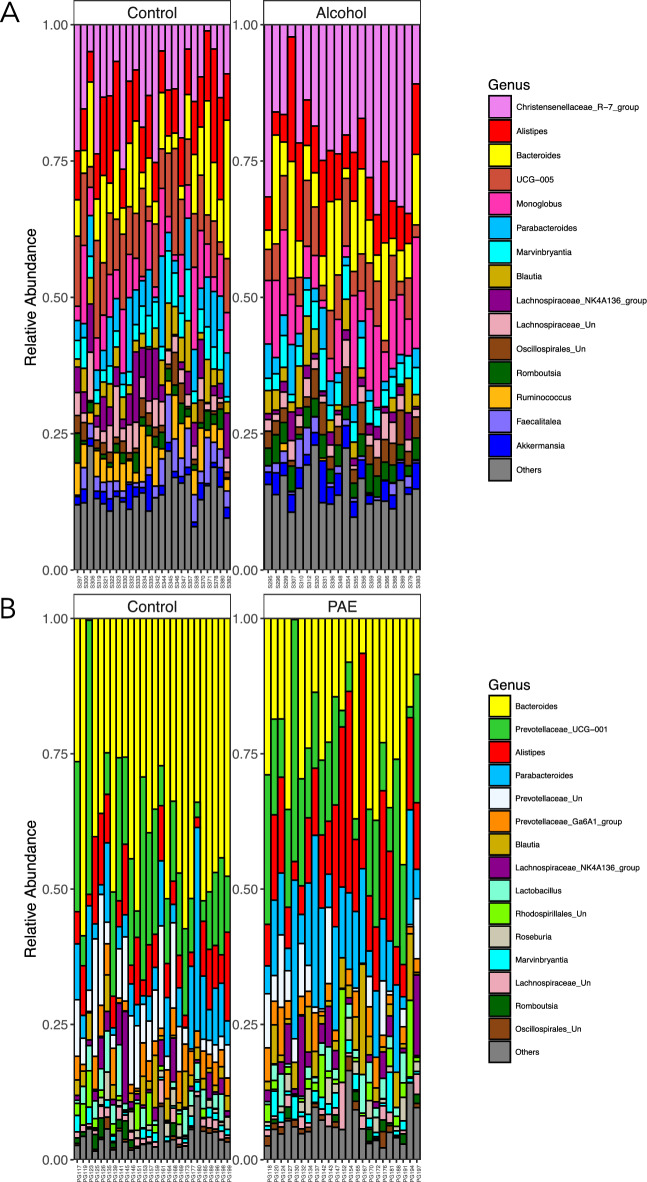
Relative abundance of bacterial taxa. Relative abundance plots of the 15 most abundant bacterial genera for dams (**A**) and offspring (**B**). All other genera shown as other (grey). *PAE* prenatal alcohol exposure. A dams: n = 20; C dams = 24; PAE offspring: n = 23; C offspring: n = 26.

Next, the relative abundance of the top 15 most abundant bacterial genera was plotted as a visual representation of the bacterial taxa changes associated with alcohol consumption during pregnancy and PAE. The relative abundance plots clearly showed that dam and offspring taxa composition were different from each other (Fig. 3). In the dams, alcohol consumption resulted in an increased abundance of *Romboutsi*a and a decreased abundance of *Lachnospiraceae NK4A136 group* and *Ruminococcus* (including a dramatic decrease in abundance or loss of *Ruminococcus* in the majority of dams that consumed alcohol; Fig. 3A, Supplementary Materials). By contrast, in the offspring, PAE resulted in an increased abundance of *Parabacteroides* and in *Alistipes* (Fig. 3B).

**Figure 4:**
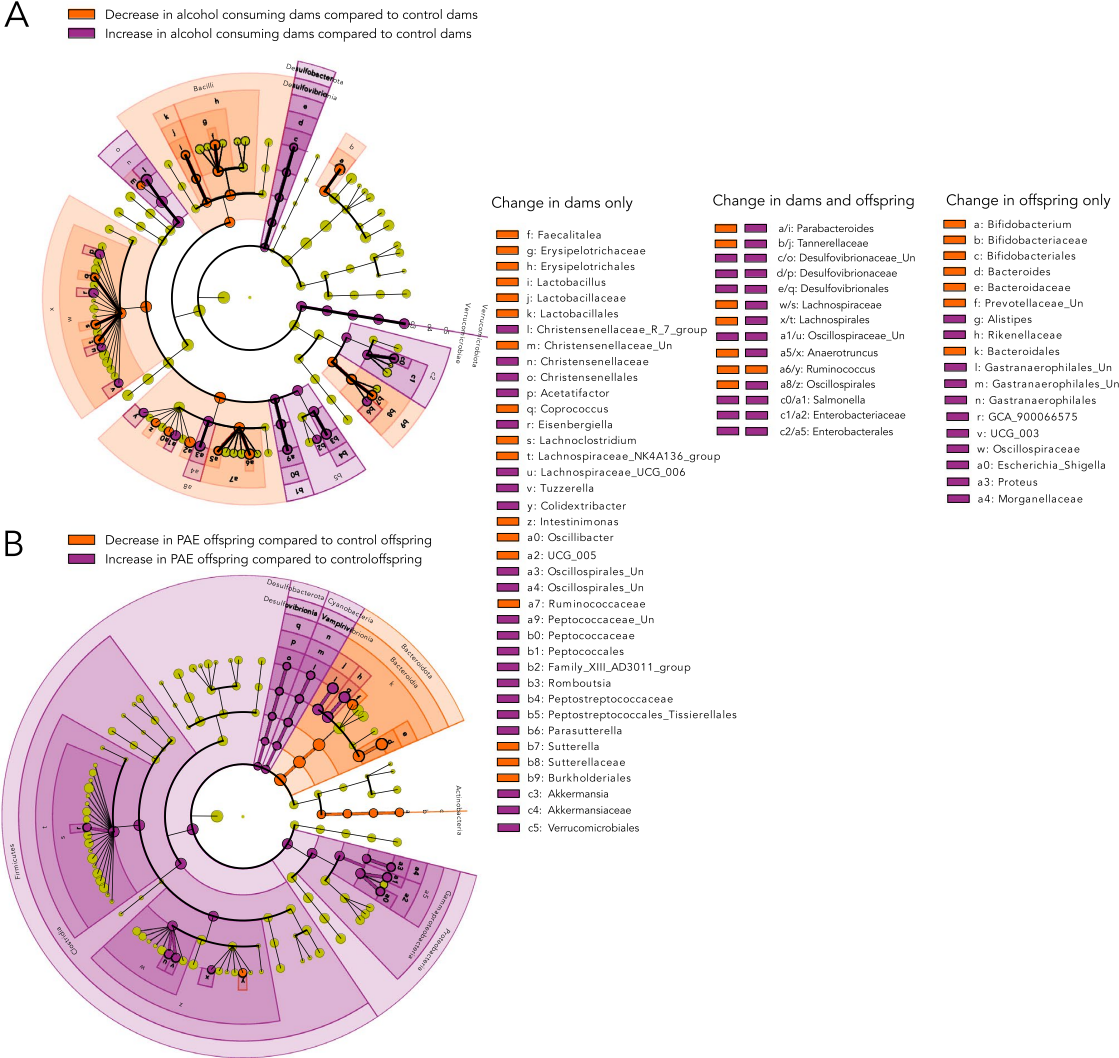
Impact of alcohol consumption and PAE on bacterial clades. Linear discriminant analysis of effect size (LEfSe), with the cladogram representing the complete taxonomy (from phylum to species). Purple shading indicates a taxon that is enriched in alcohol consuming dams (**A**) or PAE offspring (**B**), whereas orange shading indicates a taxon that is decreased in alcohol consuming dams (**A**) or PAE offspring (**B**). Yellow circles indicate a taxon that does not differ in relative abundance between treatments. A alcohol, PAE prenatal alcohol exposure, C control, A dams: n = 20; C dams = 24; PAE offspring: n = 23; C offspring: n = 26.

Following the detection of community level changes due to alcohol consumption during pregnancy and PAE, LEfSE analyses were conducted to determine which bacterial taxa were driving these differences (Fig. 4). For the dams, LEfSE showed that a total of 57 taxa (all ranks cofounded) were increased (32) or decreased (25) with alcohol consumption during pregnancy. Interestingly, alcohol consumption during pregnancy resulted in a general increase in aerotolerant bacteria Desulfovibrionaceae, Akkermansia (Verrucomicrobiota) and the order Enterobacteriales, which has been linked to intestinal inflammation^23^. In addition, the majority of differences in the alcohol consuming dams were detected at lower taxonomic levels (specific changes of Families and Genera) (Fig. 4A). For the offspring, a total of 43 taxa (all ranks cofounded) were increased (32) or decreased (11) with PAE. In contrast to the dams, taxa differences in PAE offspring were generally detected at higher taxonomic levels (i.e. broad changes of at the Phyla and Class level). In addition, PAE offspring showed an increase in the phylum Firmicutes, as well as a decrease in *Ruminococcus, *which was also detected in the dams (Fig. 4). PAE also resulted in a general decrease in the phylum Bacteroidota*,* with decreases in the genus *Bacteroides* and an unclassified clade in the Prevotellaceae*,* though the genera *Parabacterteroides* and *Alistipes* were increased in PAE compared to C offspring (Fig. 4B). Interestingly, the order Enterobacterales was found to increase in PAE offspring, echoing the increase also detected in alcohol-consuming dams. This increase was driven by increased relative abundance of *Salmonella*, a well-known pathogenic genus, in both alcohol consuming dams and PAE offspring (Fig. 4). DESeq2 analyses also support the findings of LEfSe, though fewer taxa differed significantly (Supplementary Materials). Finally, the ratio of Lactobacillus to Enterobacteriaceae, a marker of gut health^24^, was calculated. This ratio was significantly lower in both alcohol-consuming dams (ratio: 0.3), compared to control dams (ratio: 16.8; U = 391, p = 0.00009), and PAE offspring (ratio: 42.8), compared to control offspring (ratio: 212.3; U = 318, p = 0.01).

### Comparisons of the overlap in shared amplicon sequence variants (ASVs) between dam and offspring showed significant differences by treatment group

**Figure 5:**
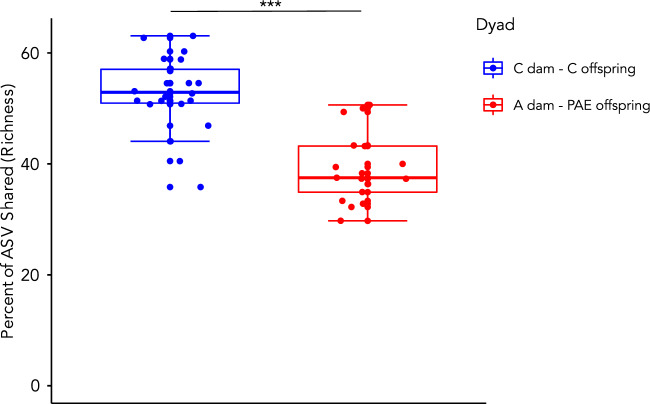
Comparison of the overlap in shared ASVs between dyads. The percentage of ASVs that were shared between dam and offspring dyads, by treatment group. ***p < 0.001. A alcohol, PAE prenatal alcohol exposure, C control, Control dam—control offspring dyads: n = 22; Alcohol-consuming dam—PAE offspring: n = 17.

To examine whether treatment influenced the degree of microbial transmission between dam and offspring and longer-term colonization, the percentage of ASVs that were shared between dams and offspring dyads was calculated (Fig. 5). Alcohol-consuming dams and their PAE offspring were found to share significantly fewer ASVs, as compared to control dams and offspring t(35.5) = 6.53, p = 0.0000001).

### Alcohol consumption during pregnancy and PAE affected the predicted biochemical pathway composition, with minimal overlap in the pathways impacted in alcohol-consuming dams and PAE offspring

**Figure 6:**
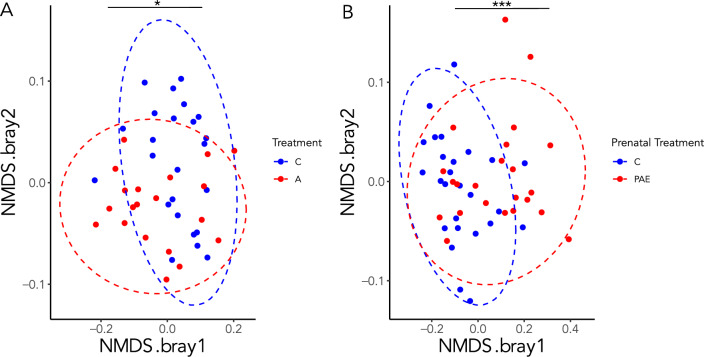
Predicted metabolic pathway composition (β-diversity). Non-metric multidimensional scaling (NMDS) plots of Bray–Curtis dissimilarity in two dimensions with ellipsoids representing 95% confidence intervals for dams (**A**) and offspring (**B**). *p < 0.05; ***p < 0.001; A alcohol, PAE prenatal alcohol exposure, C control; A dams: n = 20; C dams = 24; PAE offspring: n = 23; C offspring: n = 26.

To examine the impact of alcohol on the predicted fecal metabolic pathway composition, we calculated the Bray–Curtis dissimilarity in pathways as predicted by PiCrust. For dams, alcohol consumption during pregnancy resulted in distinct clustering compared to that in controls (Fig. 6A) and this was supported by a significant PERMANOVA (Pseudo-F = 3.51, df = 1, R^2^ = 0.08, p = 0.03). Similarly, distinct clustering was observed between PAE and C offspring, and supported by a significant PERMANOVA ((Pseudo-F = 13.4, df = 1, R^2^ = 0.22, p = 0.001); Fig. 6B).

**Figure 7:**
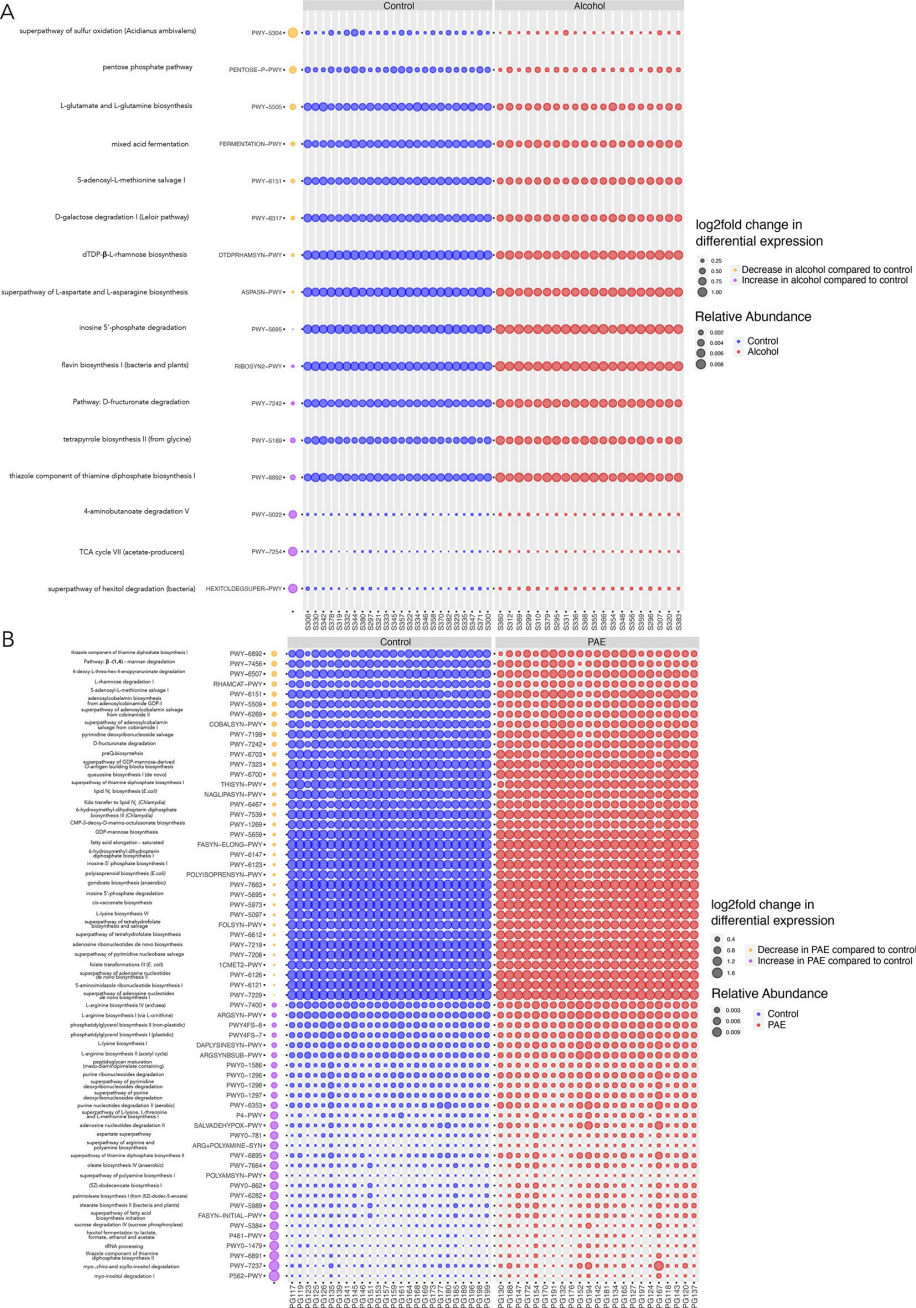
Differential abundance between treatments in predicted metabolic pathways. DESeq2 identifying predicted pathways that were significantly different with alcohol consumption (**A**) or exposure (**B**). Orange dots indicate decreases and purple dots indicate increases in alcohol consuming dams compared to controls (**A**) or PAE offspring compared to controls (**B**), respectively, with the size of the dot being proportional to the log2fold change in differential expression. Blue (controls) and red (alcohol consuming or PAE) dots represent the relative abundance of each pathway, for animals in which it was present. A and PAE samples were ordered based on maternal average alcohol consumption from GD7–14 (highest on the left to lowest on the right). A alcohol, PAE prenatal alcohol exposure, C control; A dams: n = 20, C dams = 24; PAE offspring: n = 23; C offspring: n = 26.

Next, differential abundance analyses were conducted using DESeq2 to probe for differences in the predicted metabolic pathways between treatments. For the dams, alcohol consumption resulted in an overall decrease in 8 pathways, with the greatest decrease in PWY-5304 (superpathway of sulfur oxidation (*Acidianus ambivalens*)) and an overall increase in 8 pathways, with the greatest increases in HEXITOLDEGSUPER-PWY (superpathway of hexitol degradation (bacteria)) and PWY-7254 (TCA cycle VII (acetate-producers); Fig. 7A). By contrast, in the offspring, PAE resulted in noticeably more changes in pathways (Fig. 7B). Specifically, PAE offspring showed a decrease in 35 pathways, with the largest decrease in PWY-6892 (thiazole component of thiamine diphosphate biosynthesis I) and an increase in 28 pathways, with the greatest increases in P562-PWY (*myo*-inositol degradation I) and PWY-7237 (*myo*-, *chiro*- and *scyllo*-inositol degradation). Interestingly, there were only two pathways, PWY-6151 (*S*-adenosyl-l-methionine salvage I) and PWY-5695 (inosine 5ʹ-phosphate degradation), that were similarly affected (decreased) between alcohol-consuming dams and their offspring. In addition, two pathways showed opposing effects in alcohol-consuming dams and their offspring. PWY-7242 (D-fructuronate degradation) was increased in dams and decreased in offspring, whereas PWY-6892 (thiazole component of thiamine diphosphate biosynthesis I), which was the pathway that showed the greatest decrease in PAE offspring, was found to be *increased* in alcohol-consuming dams. Overall, alcohol-consumption was found to result in larger fold-changes in pathways that *increased,* as compared to decreased, with the opposite pattern detected in PAE offspring.

## Discussion

The current study explored the impact of maternal alcohol consumption and in utero alcohol exposure on fecal microbiota composition. Bacterial richness (α-diversity) was decreased in alcohol-consuming dams, compared to their control counterparts, with no differences detected between PAE and control offspring. By contrast, community structure (β-diversity) was altered by both alcohol-consumption and PAE, with distinct clustering by treatment. Importantly, relative abundance analyses visually showed that the community composition of dams and offspring differed greatly. Subsequently, differential abundance analyses showed that alcohol-consumption and PAE resulted in numerous changes to specific bacteria at various taxonomic levels. Interestingly, there was very little overlap between alcohol-consuming dams and PAE offspring, in terms of the taxa driving the differences in community composition. For example, phylum level changes to Firmicutes, Bacteroidota, Cyanobacteria, and Proteobacteria were only observed in PAE offspring, while changes to the phylum Verrucomicrobiota were unique to alcohol-consuming dams. Similarly, alcohol consumption and PAE were found to differentially impact biochemical pathway composition, with more pathways found to be altered in PAE offspring than in alcohol-consuming dams. Finally, this study is unique in exploring microbiotal changes induced by alcohol consumption in dam-offspring dyads. Interestingly, alcohol consuming dams and their PAE offspring were found to have fewer shared ASVs than control dyads. Taken together, these results highlight the unique effects of alcohol consumption and PAE on the fecal microbiota and point to the importance of future investigation of the role of microbiotal changes in mediating the physiological and behavioral alterations induced by PAE.

The finding of decreased bacterial diversity with alcohol consumption is generally supported by studies on alcohol consumption in adult rodents^25^. However, microbiotal changes are also known to occur during pregnancy in response to hormonal changes, and acting synergistically with immune changes, help protect the fetus and support pregnancy-related physiological processes^26^. Overall, bacterial diversity has been shown to decrease with healthy pregnancy (reviewed in^18^). Thus, the current findings that alcohol-consuming dams showed decreased diversity compared to their pregnant control counterparts indicate an exaggerated reduction in diversity. Similar patterns of reductions in bacterial diversity during pregnancy have been observed in models of other adverse exposures (e.g., antibiotic exposure^27^) and in various health conditions (e.g. inflammatory bowel disease^28^). By contrast, attenuated decreases in bacterial diversity during pregnancy have been detected in individuals consuming a healthy diet and this is associated with a reduced likelihood of adverse perinatal outcomes^29^. In addition, it is interesting that, in this study on weaning age offspring (P22), we did not detect an impact of PAE on bacterial diversity, in contrast to our previous work in which an *increase* in diversity was detected in adulthood (P90)^11^. Indeed, the microbiota has been shown to mature during early development, with lower diversity around weaning, compared to that in the adult organism^30^. Thus, it appears that the impact of PAE on diversity may emerge as the organism develops. Finally, both alcohol consumption during pregnancy and PAE impacted bacterial community structure, which is in line with our findings in adult PAE animals^11^, as well as data from other models of prenatal exposures^31,32^.

At the taxa level, alcohol consuming dams showed decreases in many of the dominant classes within the phylum Firmicutes (e.g. Bacilli, and Erysipelotrichia), as well as both increases and decreases in many members of the class Clostridia (e.g. members of the family Ruminococcaceae were decreased and members of the family Peptococcaceae were increased). Of relevance, decreases in the *Lactobacilli* genus and increases in the Enterobacteriaceae family were detected in alcohol-consuming dams. Lactobacilli are known to play a beneficial role in gut health^33^, with lower levels detected in many disease states (reviewed in^34^). Previous work has shown that *Lactobacilli* limit the proliferation of pathogenic bacteria within the family Enterobacteriaceae through the production of lactic acid and a subsequent decrease in pH^35,36^. By contrast, alcohol-induced increases in fecal pH likely decrease *Lactobacilli* and concomitantly allow for increases in Enterobacteriaceae^24^. Furthermore, Enterobacteriaceae were also increased in PAE offspring and this may be a key driver of our previously reported PAE-induced exacerbations in inflammation^37,38^, as Enterobacteriaceae blooms have been linked to inflammation and gut dysbiosis^39^. Finally, the ratio between Lactobacilli and Enterobacteriaceae was also decreased in both alcohol-consuming dams and PAE offspring, compared to their control counterparts, indicative of poor gut health^24^. Taken together, while further research is needed, the observed increase in Enterobacteriaceae points to possible benefits of *Lactobacillus*-containing probiotic administration in the current model.

Alcohol-consuming dams also showed an overall decrease in the families Ruminococcaceae and Lachnospiraceae, which are known to include the majority of butyrate producing microbes in the gut^40^. Importantly, there is growing evidence that butyrate is neuroprotective^41^, exerts anti-inflammatory effects^42,43^, and is critical for gut barrier function and gut health^44^. In addition, there is also evidence for possible developmental impacts of alterations in prenatal butyrate levels. For example, an Australian birth cohort study has linked an increased abundance of maternal butyrate producers (Lachnospiraceae and Ruminococcaceae) with typical behaviors (decreased child internalizing behaviors at age 2)^45^. Prenatal butyrate supplementation in rodents has also been linked to beneficial impacts on the offspring including decreased inflammation-associated colonic gene expression and improved outcomes in a colitis model^46^. Thus, it stands to reason that alcohol-induced decreases in maternal butyrate could be driving, at least in part, some of the adverse effects of PAE such as increased levels of pro-inflammatory cytokines^37^ and the alterations in behavior that we have previously identified in this model^47,48^. Alcohol-consuming dams and PAE offspring also had an increase in the family Desulfovibrionaceae. Of relevance, increases in Desulfovibrionaceae have also been identified as a signature of intestinal dysbiosis^49^. Desulfovibrionaceae are known for their ability to reduce sulfate and produced toxic hydrogen sulfide, which likely impacts disulfide bonds in the intestinal epithelium, impairing barrier function and leading to inflammation^50^. As such, Desulfovibrionaceae may be another important downstream driver of our previously reported increase in inflammation in PAE offspring^37,38^. Within Desulfovibrionaceae*,* the genus *Desulfovibrio* has also been shown to be increased in children with ASD^51,52^, with likely effects on gut physiology due to their production of propionic acid (PPA). PPA has a wide range of effects on gut physiology including decreasing gastric motility and is linked to gastrointestinal abnormalities in children with ASD (reviewed in Ref.^53^). In addition, it has been shown that elimination of PPA-producing bacteria through antibiotic administration may improve behavior and gastrointestinal functioning in ASD^53,54^, and this may also warrant further investigation in FASD.

There is growing evidence that interactions between the gut microbiota and the central nervous system are critically important for brain development and subsequently behavior^6,55,56^. Importantly, in the current study, PAE was shown to alter the abundance of a number of taxa that have been demonstrated to impact behavior. For example, PAE animals had a decreased abundance of the family Bifidobacteriaceae, which are known to be critically important in the infant gut^57^, playing a protective role against pathogens and with decreased abundance associated with disease^57^. Bifidobacteria also exert neuromodulatory effects and have been shown to drive CNS alterations during important periods of postnatal brain development^55^. PAE also resulted in an increased abundance of the genera Alistipes and Parabacteroides within the order Bacteroidales. Alistipes*,* a relatively new genus of bacteria, has recently been implicated in inflammation and disease^58,59^. In addition, there is emerging evidence that increased Alistipes abundance may negatively impact mental health, with stress exposure linked to increased Alistipes abundance in a rodent model^60^ and depression in a clinical sample^61^. Similarly, Parabacteroides is correlated with depressive-like behaviors in rodents, with administration of a single species of *Parabacteroides* into specific pathogen-free mice (model of Crohn’s disease) shown to induce depressive-like behaviors^62^. While more research is needed to understand the link between the gut microbiota and mental health, including depression, this may be a promising area for consideration in FASD as mental health problems occur at disproportionately high rates in this population^63^.

Both alcohol-consumption and PAE were also shown to impact predicted metabolic pathway composition in the current study. Differential abundance analyses showed that 8 pathways were increased and another 8 pathways were decreased in alcohol-consuming dams, whereas, by contrast, 28 pathways were increased and 35 pathways were decreased in PAE offspring. This suggests that PAE resulted in a more substantial impact on metabolic pathways than adult alcohol-consumption. In addition, examination of pathway functions indicated greater diversity in the pathways that were altered in the PAE offspring, compared to the alcohol-consuming dams (i.e. wider range of functions impacted by PAE). Of the pathways altered in PAE offspring, overall functions included: amino acid synthesis (DAPLYSINESYN-PWY, P4-PWY, PWY0-781), fatty acid biosynthesis (FASYN-INITIAL-PWY), carbohydrate degradation (P461-PWY, PWY-6507, PWY-7242, PWY-5384), LPS biosynthesis (PWY-7323, NAGLIPASYN-PWY, PWY-6467, PWY-1269, POLYISOPRENSYN-PWY), nucleoside synthesis (PWY-6121, PWY-7199, PWY-7208), vitamin biosynthesis (PWY-6891), and inositol degradation (P562-PWY, PWY-7237). Previous work has shown developmental changes in the functional potential of the gut microbiome in juveniles compared to adults^64^. And notably, in the current study, a number of pathways that would be expected to be enriched at the weanling (P22) stage were decreased in PAE offspring. For example, pathways involved in cobalamin synthesis, which has important anti-inflammatory effects and is critical for brain function^65^, and de novo folate synthesis, which is key for DNA synthesis, replication and repair^66^, were decreased in PAE offspring. Importantly, there was also minimal overlap between the pathways that were altered by alcohol in the dam and offspring. In fact, there were only four overlapping pathways (inosine 5ʹ-phosphate degradation, *S*-adenosyl-l-methionine salvage I, thiazole component of thiamine diphosphate biosynthesis I, and d-fructuronate degradation) with only two pathways affected in the same direction (*decrease* in the *S-*adenosyl-l-methionine salvage I pathway and the inosine 5ʹ-phosphate degradation pathway in both PAE offspring and alcohol-consuming dams). The observed metabolic shift in the S*-*adenosyl-l-methionine salvage I pathway is noteworthy as this may have implications for the known epigenetic changes that occur following PAE^67,68^. In addition, it has been shown that bacterial folate biosynthesis may play an important role in epigenetic regulation during development^69^. However, to the best of our knowledge, the link between the gut microbiota and epigenetic changes has yet to be explored following PAE. Finally, while additional work will be required to confirm and extend our understanding of the impact of alcohol on metabolic pathways, including direct measurements of metabolites, the extensive and wide-ranging impact of PAE, in particular, on these predicted metabolic pathways suggest likely functional consequences.

Finally, a goal of the current study was also to compare the impact of alcohol consumption on the microbiota of the dam to that of her offspring in order to assess whether changes to the maternal microbiota would be transmitted to the offspring. In other words, assessing whether maternal alcohol consumption impacted vertical transmission to her offspring. This was assessed through comparing the percentage of shared ASVs between dam and offspring dyads. Interestingly, alcohol-consuming dams were found to have fewer shared ASVs with their offspring, as compared to controls dyads. This suggests that that vertical transmission of the microbiota may be altered with alcohol consumption and that the source of bacterial diversity in PAE offspring stems from other non-maternal fecal microbiome sources.

### Limitations of the study

The results of the current study indicate that both alcohol-consumption and PAE have an impact on the microbiota; however, there is very limited research in this area and more work is needed in order to fully understand the impact of PAE on the microbiota. There are also some limitations to the current study. First, while the pelleted and liquid diets were isocaloric and the composition and nutrient profile of the pelleted and liquid diets administered were matched, cornstarch is added to the pelleted diet to facilitate pelleting (see Supplementary Materials for details). In other experimental paradigms (e.g., high fat and obesity models), larger amounts of cornstarch has been shown to impact the gut microbiota^70^; however, cornstarch is also a relatively common component of regular laboratory chow^71^. In the current model, a nutritionally comparable pelleted control diet was selected over an ad libitum-fed liquid control diet for our control group, as we have previously shown that ad libitum*-*fed liquid diet increased diet consumption and maternal weight gain^72^, with likely consequences for the microbiota^73,74^. Nevertheless, the nutritional profile of the diet in microbiota research is clearly critical and warrants further investigation and standardization among research groups^75^. Second, it is important to recognize that the microbiota is well known to change and mature across the lifespan^30^. As such, future developmental studies will be needed to probe for both transient dysbiosis and possible consistent, life-long microbiotal alterations characteristic of PAE offspring. To this end, it is noteworthy that Enterobacteriaceae, which contains pathogenic genera, was shown to be elevated in the current study and that our findings in adult PAE offspring indicate that this appears to persist throughout the lifecourse^11^. In addition, a possible detrimental decrease in Bifidobacteriaceae was identified in the current study, again matching our previous work on PAE adults^11^. Finally, it will be important that future work begins to establish a link between PAE-induced alterations in the microbiota and functional impacts, such as alterations in immune function/inflammation, health, and cognitive and behavioral outcomes.

### Conclusions

Taken together, the current study extends our understanding of the impact of alcohol-consumption during pregnancy on the microbiota of the dam and her offspring. Importantly, minimal overlap was detected between alcohol-induced changes in dams and offspring, highlighting that microbiotal alterations are not simply transmitted to the offspring. In addition, the identification of altered taxa, including possible pathogenic taxa and indicators of impaired gut health, as well as a wide range of metabolic pathways altered in PAE offspring strongly support the consideration of the microbiota in the pathophysiology of FASD, and signal possible novel intervention approaches.

## Methods

### Breeding

Sprague–Dawley (SD) rats (barrier room C-72, Charles River Laboratories, St. Constant, Quebec, Canada) were pair-housed by sex and handled daily for a 2-week habituation period prior to the start of the experiment. Colony rooms were maintained on a 12:12 h light/dark cycle (lights on at 0700 h), at 20–23 °C. During the habituation period, rats were provided with standard laboratory chow (18% Protein Extruded Rodent Diet, #2018, Teklad Global). Following the habituation period, nulliparous females (250 – 325 g; postnatal day 64–74; n = 53) were each housed with a male and vaginal lavage samples were collected daily to complete estrous cycle staging and to check for the presence of sperm, indicating gestation day 1 (GD1). Animals were housed in autoclaved cages with beta-chip bedding throughout the experiment. All animal procedures were in accordance with the National Institutes of Health Guide for the Care and Use of Laboratory Animals and the Canadian Council on Animal Care (CCAC) and approved by the University of British Columbia Animal Care Committee. Reporting of the data also follows the recommendations in the ARRIVE guidelines.

### Prenatal diets and feeding

On GD1, females were single housed and assigned to one of two treatment groups: (1) Prenatal alcohol exposure (PAE)—ad libitum access to an alcohol-containing liquid diet (Weinberg/Keiver High Protein Liquid Ethanol Diet—experimental. #710324) with 36% of total calories derived from ethanol, n = 20; or (2) Control (C)—pelleted version of the liquid control diet (Weinberg/Keiver High Protein Pelleted Control Diet #102698), ad libitum, n = 24. All diets were formulated to provide optimal nutrition during pregnancy (Dyets Inc. Bethlehem, PA, USA; full nutritional information is available in Supplementary Materials). Liquid diet was prepared fresh daily and presented one hour prior to lights off (1800–1900 h). To facilitate acceptance of the liquid ethanol diet, this diet was introduced gradually: on GD1, rats were provided with a 1:2 ratio of liquid ethanol to liquid control diet (Weinberg/ Keiver High Protein Liquid Control Diet#710109); on GD2, rats were provided a 2:1 ratio of liquid ethanol to liquid control diet; and on GD3 rats received only liquid ethanol diet. On GD17, a blood sample was collected from the tail vein from a subset of dams (n = 3–6/group) at lights on (0700 h). Blood was collected from the tail vein on GD14 from a subset of animals not included in the microbiome analyses to measure blood alcohol levels (BALs), as previously reported^37^. BALs ranged from ~ 80 to 150 mg/dl in PAE rats. On GD21, experimental diets were replaced with laboratory chow (19% Protein Extruded Rodent Diet, #2019, Teklad Global), ad libitum, with rats continuing on this diet throughout the lactation period. Chlorinated reverse osmosis (RO) water in sterilized bottles was provided ad libitum throughout the study. During pregnancy and lactation only, cages were equipped with filter-top lids and were maintained on ventilated racks.

### Animal rearing

On the day of birth (postnatal day 1; P1), litters were culled to six males and six females, when possible. Dams and pups were weighed weekly during the lactation period. Pups were weaned on P22, rehoused by treatment and sex, and provided with the 18% protein chow (Teklad Global #2018) ad libitum.

### Fecal sample collection and processing

Fresh fecal samples were collected into autoclaved tubes, using sterile tools, on GD14 (dams: 78–88 days old) and P22 (offspring). GD14 was selected as the dams were well established on the experimental diet by this point and parturition, and the associated inflammatory changes, would not be occurring for another week. P22 was selected in order to examine the early-post weaning microbiota; fecal collection occurred immediately prior to animals being provided with the 18% protein diet (Teklad Global #2018). The gut microbiome has been shown to exist, broadly, in two states post weaning, an early (first 10–15 days post weaning) and a late state (15 days+)^76^ and in order to better probe for a link between the microbiota of the dam and offspring, the early state was selected (i.e., P22). Fecal samples were stored at − 80 °C and shipped to the Integrated Microbiome Resource (IMR) at Dalhousie University (Halifax, Nova Scotia, Canada) for 16s rRNA sequencing^77^. PCR amplification of bacterial 16s rRNA gene-targeted the V6–V8 regions (primers—B969F: ACGCGHNRAACCTTACC and BA1406R: ACGGGCRGTGWGTRCAA). Amplicon sequencing was done using the Illumina MiSeq with 300–300 bp paired-end V3 chemistry^77^.

### Bioinformatic analysis

#### Data processing and taxonomy assignment

Dam and offspring demultiplexed raw Fastq files were separately processed using the dada2 package in R Studio (version 4.2.0)^78^. Both Fastq files were primer trimmed, quality filtered, and processed. ASVs with a relative abundance of less than 0.001 were removed to filter out singletons and low read counts for each sample within the dada2 pipeline. The dada2 pipeline was also used to identify and remove chimeras. After the initial processing, the dam and offspring sequences were merged to assign taxonomy. Taxonomy was assigned within the dada2 pipeline using the SILVA 138 database^79^. Post dada2 processing was done separately for the dam and offspring datasets using the phyloseq package. ASVs identified as chloroplast, mitochondria, and eukaryotic were filtered out, as well as sequences unassigned at the domain level. ASVs with counts less than two per sample were also filtered out to minimize the potential effects of barcode switching. In addition, low sequence ASVs with less than 250 total sequences were filtered out. For community level analyses, the datasets were rarefied to the minimum ASV count (in order to prevent sample loss): 3029 for the dam and 20,042 for offspring, respectively. The average read depth was 29,623 for the dam and 37,996 for the offspring. One control female offspring was removed as it did not contain any ASV’s. After filtration, the two separate datasets were merged into a final non-rarefied dataset for non-community level analyses (DESeq2)^80^. Dam (294 unique ASVs) and offspring (216 unique ASVs) filtered and rarefied datasets were also merged into a final rarefied dataset for both community-level (alpha and beta diversity) and non-community level analysis (LEfSe)^80^. Both merged datasets consisted of 378 unique ASVs, 102 samples (53 dam and 49 offspring), and seven taxonomic ranks. For non-community level analyses, the dam and offspring biochemical pathway datasets were rarefied to the minimum ASV count per sample of 3029 (dam) and 20,042 (offspring).

#### Biochemical pathways annotation

Biochemical pathways identified from the non-rarefied dataset were annotated using the PICRUSt2 methods (version 2.3.0 beta; Douglas et al., 2020) and using an available GitHUB pipeline (https://github.com/picrust/picrust2/wiki/PICRUSt2-Tutorial-(v2.3.0-beta)). Filtering the predicted biochemical pathway phyloseq object was conducted by removing ASVs with counts less than two per sample to minimize the potential effects of barcode switching and by removing low sequence ASVs with less than 250 total sequences. For community-level analyses, the predicted biochemical pathway dataset was rarefied to the minimum ASV count per sample of 286,508.

### Statistical analysis

Statistical analyses and visualizations were done using R Studio (version 4.2.0) using ggplot2 or the base plot package with the exception of Linear discriminant analysis of Effect Size (LEfSe), conducted through the Galaxy/Hutlab’s online web tool. All scripts are available on the Parfrey lab Github (https://github.com/parfreylab). Statistical analyses were conducted separately for dam and offspring, with *t*-tests or one-way ANOVAs being conducted for normally distributed data and Mann–Whitney U test for non-normally distributed data. P-values were adjusted for multiple comparisons using the Bonferroni procedure. For the offspring, two-way ANOVA and two-way PERMANOVA were also used to explore the interaction of prenatal treatment and sex (see Supplementary Materials). In order to calculate the ratio of Lactobacillus to Enterobacteriaceae, the data was transformed by adding one to the total number of ASV for each taxon.

#### α and β diversity

To assess the treatment effects on the dam and offspring fecal bacterial taxa and pathway diversity, α- diversity was quantified using the Shannon index (which measures both evenness and richness)^81,82^. Extreme outliers were identified as values above 3 + 3 × IQR or below Q1—3 × IQR ((Q = quartile, IQR = interquartile range) and removed (n = 1 PAE offspring and n = 1 C offspring).

Analysis of β-diversity was used to assess the treatment effects on the fecal bacterial taxa and pathway composition. β-diversity was quantified using the Bray–Curtis dissimilarity index (presence and absence-based)^83,84^ and analyzed by one-way PERMANOVA with 999 random permutations to analyze multiple samples. Non-metric multidimensional scaling (NMDS) plots of Bray–Curtis dissimilarity were used to visualize the differences in community composition, and 95% confidence intervals were illustrated as ellipsoids surrounding each group and an asterisks marking statistical significance between groups. The vegan package was used to estimate diversity indexes and perform PERMANOVA.

#### Differential taxa and pathway abundance

Relative abundance plots of the top 15 bacterial genera were used to visualize differences in taxa composition (see Supplementary Materials for relative abundance plots averaged across groups). The relative abundance of each taxon was calculated as a percentage of total abundance for each sample, grouped by treatment. Relative abundance plots were made using ggplot2. Linear discriminant analysis of Effect Size (LEfSe)^85^ was conducted to identify differentially abundant bacterial taxa between groups from the phylum to the genus level. LEfSe analysis was conducted through the Galaxy/Hutlab’s online web tool (accessed June 2022). The DESeq2 package was also used to further identify taxa that were impacted alcohol (see Supplementary Materials). Additionally, the DESeq2 package was used to identify predicted biochemical pathways that were altered by treatment. LEfSe and DESeq2 plots were color corrected using Inkscape^86^.

#### Differential ASVs between dams and offspring

The percentage of ASVs that were shared between dam and offspring dyads, by treatment group, was explored using published GitHub scripts^87^. An ASV was considered to be shared if it was present in the dam and at least one of her offspring (male or female). This resulted in the following sample size: Control dam—control offspring dyads: n = 22; Alcohol-consuming dam—PAE offspring: n = 17.

### Supplementary Information

Supplementary Figures.

Supplementary Tables.

## Data Availability

The data that support the findings of the study are available through the European Nucleotide Archive (ENA) under accession number PRJEB54795. All scripts are availasxsble on the Parfrey lab Github (https://github.com/parfreylab).
