# Supplementary material for: Alcohol consumption during pregnancy differentially affects the fecal microbiota of dams and offspring
Source: Sci Rep. 2024 Jul 12;14:16121. doi: 10.1038/s41598-024-64313-z (PMC11245617; doi:10.1038/s41598-024-64313-z)
Supplement: Supplementary file 1 — Supplementary Figures. [file 41598_2024_64313_MOESM1_ESM.pdf]

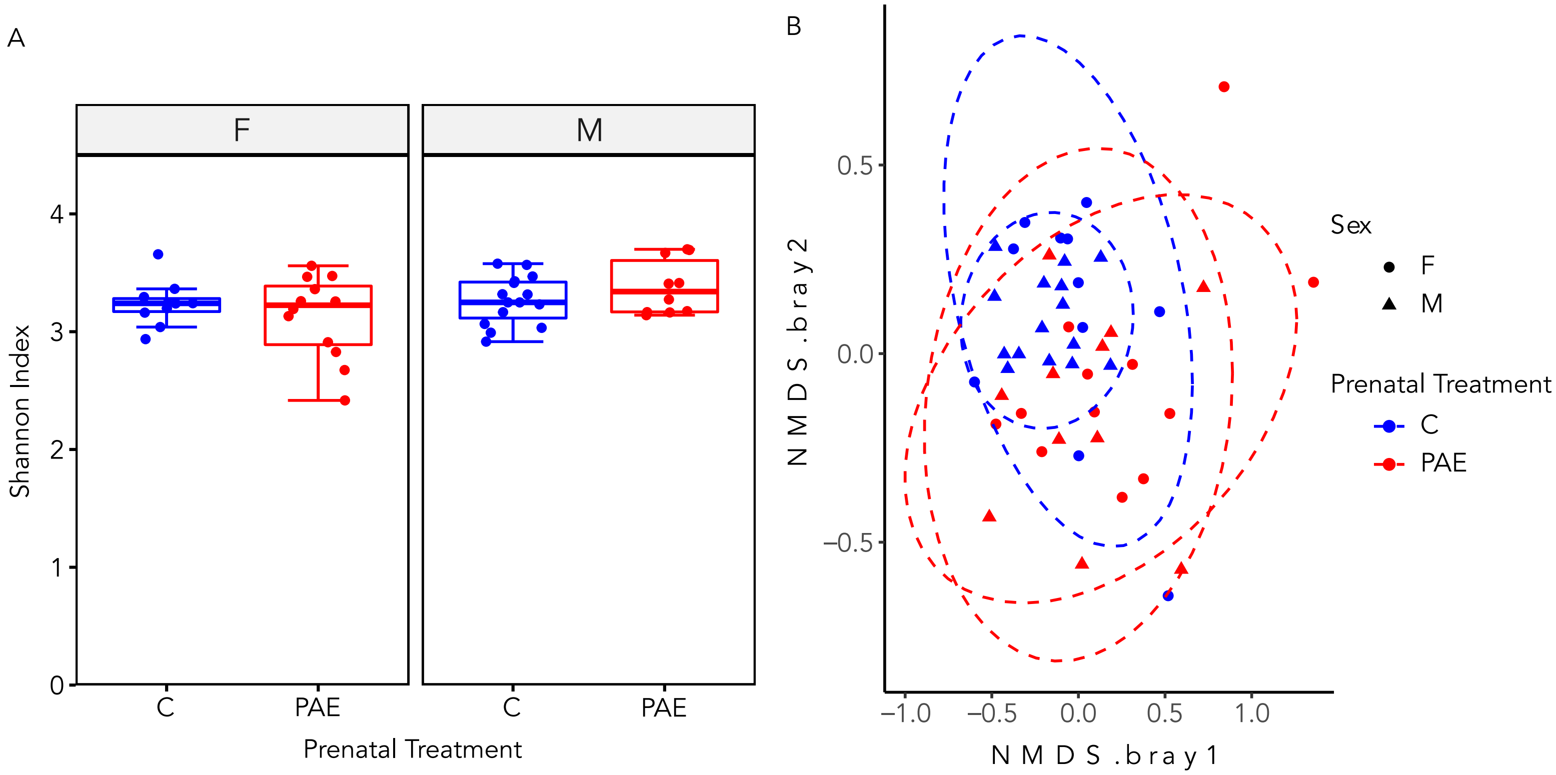

Figure S1. Lack of sex differences in the effects of prenatal alcohol exposure on bacterial α-diversity and β-diversity.
Boxplots displaying the mean Shannon index by sex (α-diversity). Points beyond the box whiskers represent extreme outliers. PAE offspring n=22, C offspring n = 25. (A).
Bray-Curtis non-metric multidimensional scaling (NMDS) plots in two dimensions with ellipsoids representing 95% confidence. PAE offspring n=23, C offspring n = 26. (B).
PAE: prenatal alcohol exposure; C: control.

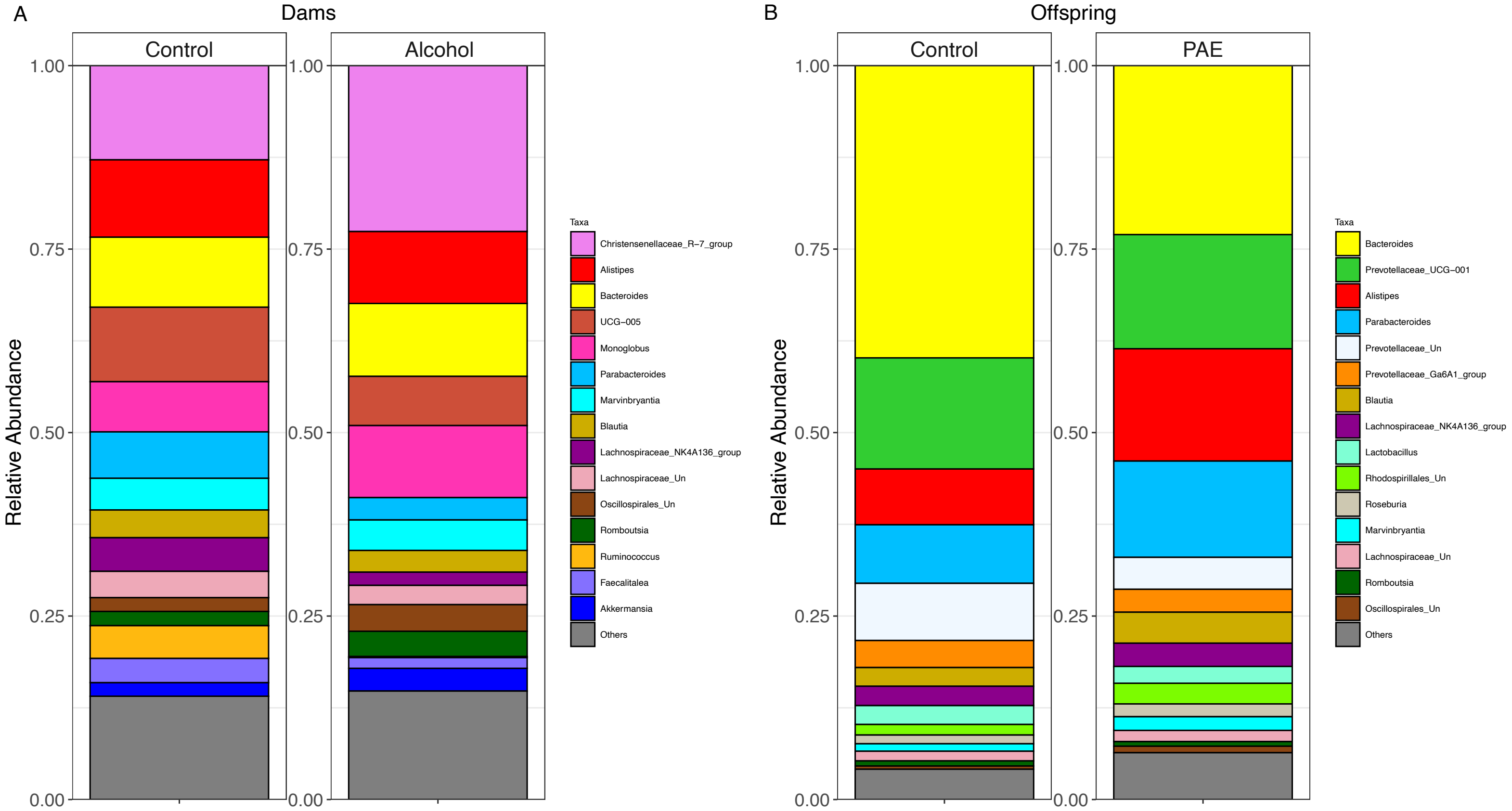

Figure S2. Relative abundance of bacterial taxa averaged across treatment groups.
Relative abundance plots of the 15 most abundant bacterial genera by treatment groups for dams (A) and offspring (B). All other genera shown as other (grey).
PAE: prenatal alcohol exposure.
A dams: n=20; C dams=24; PAE offspring: n=23; C offspring: n=26.

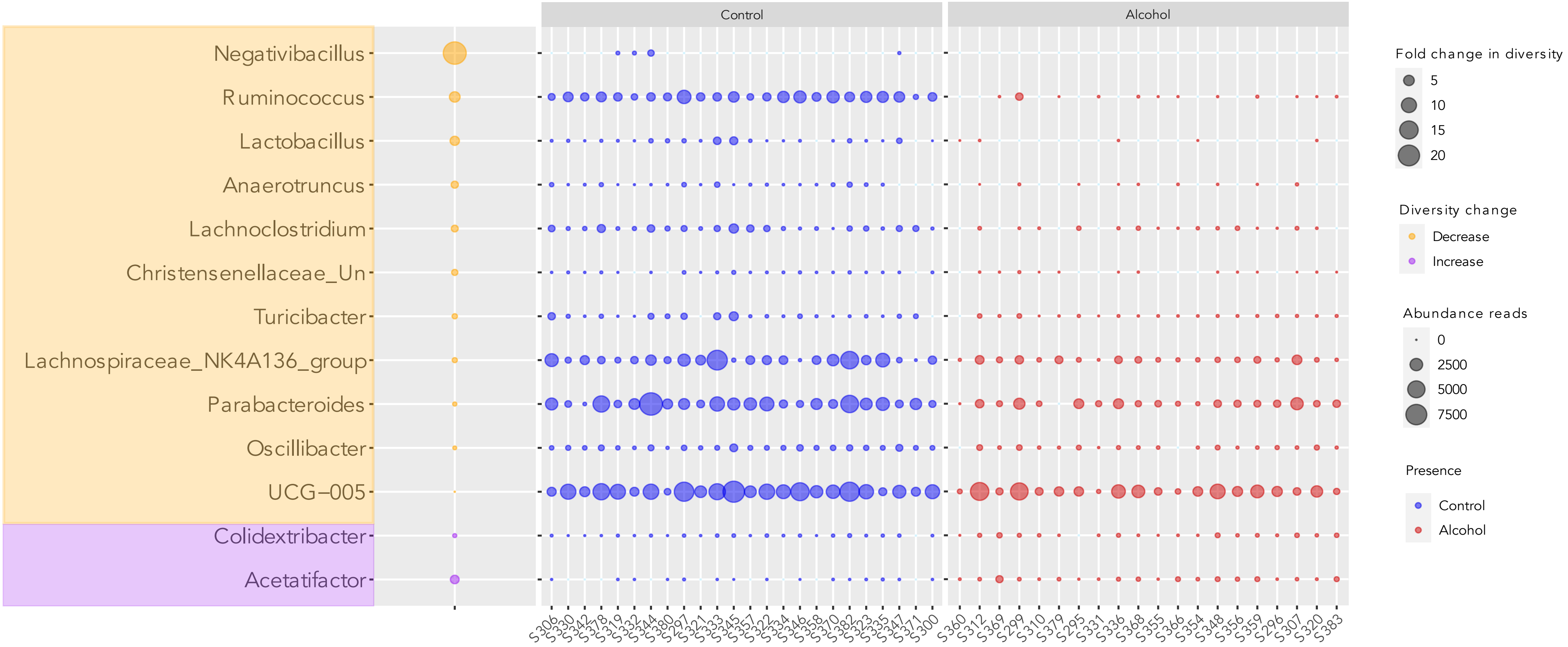

Figure S3. Differential abundance analyses to examine differences in taxa between alcohol and control dams Left: DESeq2 results of significant taxa differences between treatments. Color of the dot indicates increase or decrease in alcohol compared to control dams. Size of the dot is proportional to the log2fold change in diversity. Right: Bubble plot indicating the presence and abundance of reads for each taxon across samples as indicated by their sample ID. Samples were ordered based on average maternal alcohol consumption from GD7-14 (highest on the left to lowest on the right).
A: alcohol; C: control; A dams: n=20, C dams: n=24.

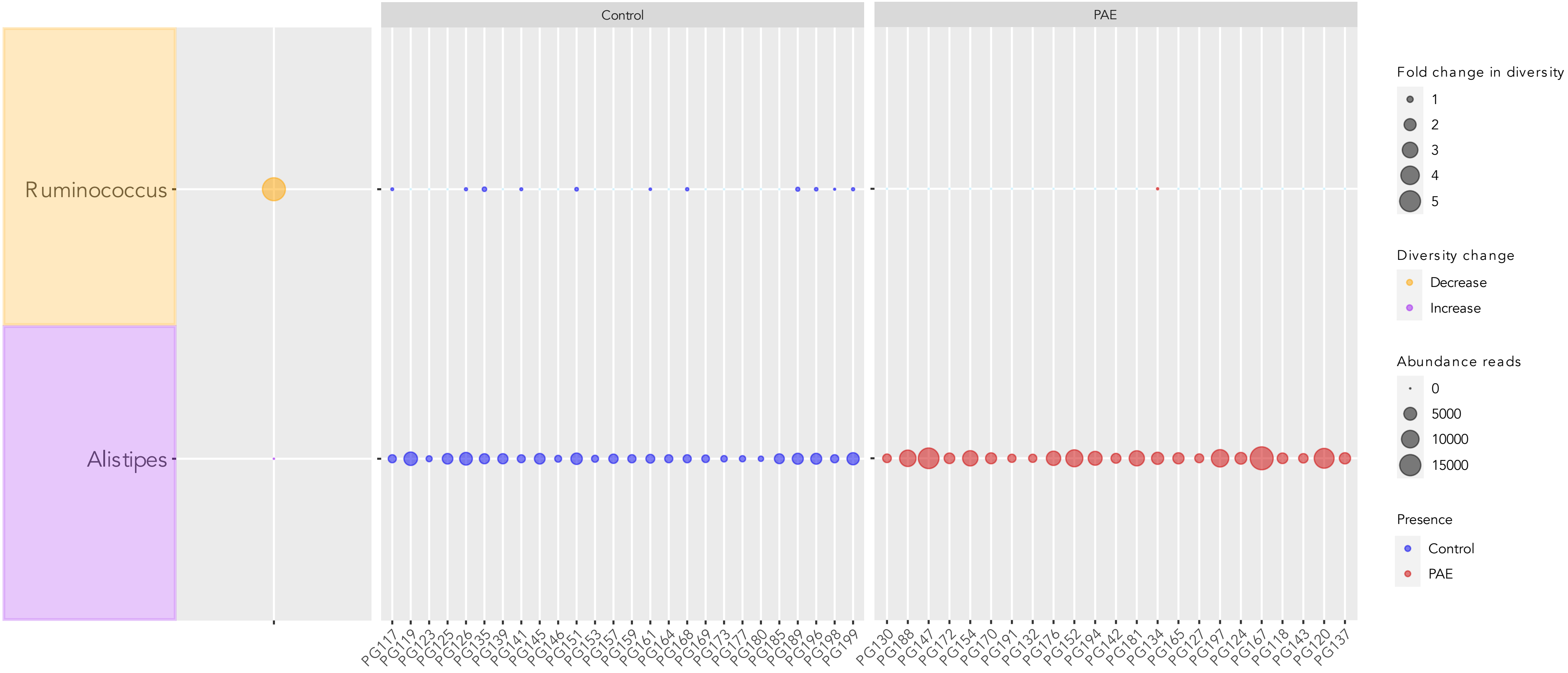

Figure S4. Differential abundance analyses to examine differences in taxa between PAE and control offspring. Left: DESeq2 results of significant taxa differences between treatments. Color of the dot indicates increase or decrease in PAE compared to control offspring. Size of the dot is proportional to the log2fold change in diversity. Right: Bubble plot indicating the presence and abundance of reads for each taxon across samples. Samples were ordered based on average maternal alcohol consumption from GD7-14 (highest on the left to lowest on the right).
PAE: prenatal alcohol exposure; C: control; PAE: n=23, C: n=26.
