# Supplementary material for: Alcohol consumption during pregnancy differentially affects the fecal microbiota of dams and offspring
Source: Sci Rep. 2024 Jul 12;14:16121. doi: 10.1038/s41598-024-64313-z (PMC11245617; doi:10.1038/s41598-024-64313-z)
Supplement: Supplementary file 2 — Supplementary Tables. [file 41598_2024_64313_MOESM2_ESM.docx]

Supplementary Table 1:

| **Ingredient** | **Liquid Ethanol Diet** | **Pelleted control diet** |
| --- | --- | --- |
| Casein | 4.3 kcal/g  57.6 g/L  245.9 kcal/L | 4.3 kcal/g  234.4 g/kg  1001 kcal/kg |
| L-Cystine | 4.0 kcal/g  0.7 g/L  2.6 kcal/L | 4.0 kcal/g  2.65 g/kg  10.6 kcal/kg |
| DL-Methionine | 4.0 kcal/g  0.4 g/L  1.6 kcal/L | 4.0 kcal/g  1.6 g/kg  6.5 kcal/kg |
| Soybean Oil | 8.84 kcal/g  18.6 g/L  164.4 kcal/L | 8.84 kcal/g  75.7 g/kg  669.0 kcal/kg |
| t-Butylhydroquinone | 0 kcal/g  0 g/L  0 kcal/L | 0 kcal/g  0.015 g/kg  0 kcal/kg |
| Maltose Dextrin | 4.0 kcal/g  53.9 g/L  213.6 kcal/L | 4.0 kcal/g  116.8 g/kg  462.5 kcal/kg |
| Dyetrose^1^ | 0 kcal/g  0 g/L  0 kcal/L | 4.0 kcal/g  116.8 g/kg  462.5 kcal/kg |
| Cornstarch | 0 kcal/g  0 g/L  0 kcal/L | 4.0 kcal/g  350.7 g/kg  1388.6 kcal/kg |
| Cellulose | 0 kcal/g  10.0 g/L  0 kcal/L | 0 kcal/g  40.7 g/kg  0 kcal/kg |
| Mineral Mix #210032 | 0.9 kcal/g  8.8 g/L  7.8 kcal/L | 0.9 kcal/g  35.6 g/kg  31.7 kcal/kg |
| Vitamin Mix #310011 | 3.6 kcal/g  2.5 g/L  9.0 kcal/L | 3.6 kcal/g  10.2 g/kg  36.6 kcal/kg |
| Choline Bitartrate | 0 kcal/g  0.7 g/L  0 kcal/L | 0 kcal/g  2.7 g/kg  0 kcal/g |
| Xanthan Gum | 0 kcal/g  3.0 g/L  0 kcal/L | 0 kcal/g  12.2 g/kg  0 kcal/g |

Diet information provided by Dyets Inc. Bethlehem, PA, USA (<https://dyets.com/>)

^1^Dyetrose: Proprietary dextrinized cornstarch that is used to help pelleting of the diet.
